# Myoferlin disturbs redox equilibrium to accelerate gastric cancer migration

**DOI:** 10.3389/fonc.2022.905230

**Published:** 2022-09-06

**Authors:** Hailong Shi, Yuanyuan Cheng, Qimei Shi, Wenzhi Liu, Xue Yang, Shuang Wang, Lin Wei, Xiangming Chen, Hao Fang

**Affiliations:** ^1^ Department of Chemotherapy, Tai’an City Central Hospital, Tai’an, China; ^2^ Department of Gastroenterology, Tai’an City Central Hospital, Tai’an, China

**Keywords:** myoferlin, gastric cancer, ROS, metastasis, GSH

## Abstract

**Objective:**

In contrast to normal cells, in which reactive oxygen species (ROS) are maintained in redox equilibrium, cancer cells are characterized by ectopic ROS accumulation. Myoferlin, a newly identified oncogene, has been associated with tumor metastasis, intracellular ROS production, and energy metabolism. The mechanism by which myoferlin regulates gastric cancer cell migration and ROS accumulation has not been determined.

**Methods:**

Myoferlin expression, intracellular ROS levels, the ratios of reduced to oxidized glutathione (GSH/GSSG) and nicotinamide adenine dinucleotide phosphate (NADPH/NADP+) and migratory ability were measured in gastric cancer cells *in vitro* and in the TCGA and GEO databases *in silico*.

**Results:**

Myoferlin was found to be more highly expressed in tumor than in normal tissues of gastric cancer patients, with higher expression of Myoferlin associated with shorter survival time. Myoferlin was associated with significantly higher intracellular ROS levels and enhanced migration of gastric cancer cells. N-acetyl-L-cysteine (NAC), a potent inhibitor of ROS, inhibited Myoferlin*-*induced ROS accumulation and cell migration.

**Conclusions:**

Myoferlin is a candidate prognostic biomarker for gastric cancer and plays an essential role in regulating redox equilibrium and gastric cancer cell migration. Myoferlin may also be a new target for treatment of patients with gastric cancer.

## Introduction

Gastric cancer (GC) is one of the top 10 malignancies worldwide, as shown by the numbers of newly diagnosed patients and deaths (1, 2). Estimates indicate that, in the United States, 26,560 patients will be newly diagnosed with gastric cancer patients and 11,180 patients will die of this disease in 2022 (3). Despite developments in chemotherapy, targeted therapy and immune therapy (4, 5), patient prognosis remains poor, especially in patients with metastatic disease. Determination of the mechanism underlying gastric cancer associated metastasis and identifying potential molecular targets may improve outcomes in patients with gastric cancer.

The ferlins are a family of proteins involved in vesicle fusion, that include FER-1, Dysferlin, Otoferlin, Myoferlin, FER1L4, FER1L5 and FER1L6 (6). Ferlins have been found to participate in processes that require membrane fusion, including endocytosis, exocytosis, and membrane repair, recycling and remodeling, membrane processes crucial for cell signaling, survival, and adaptation to hostile environments (6). Moreover, the levels of expression of several ferlins were reported to be associated with survival in patients with several types of cancer (7).

The Myoferlin gene, which is 180 kb in length and composed of 54 exons (6), encodes the Myoferlin (MYOF) protein, which consists of 2061 amino acids (8). MYOF is an endocytosis and vesicle-transport-related membrane protein made up of six C2 domains, including C2A, N-terminal-C2B-Fer1-C2C, C2D and two C-terminal C2 (C2E-C2F) domains, located close to a single transmembrane domain (8). Furthermore, the *MYOF* gene promoter contains many consensus-binding sites, including for Myc, MEF2, CEBP, Sp1, AP1, MKL1/2 and NFAT (9, 10).

MYOF has been reported to be upregulated in several types of cancer, including triple-negative breast cancer (TNBC) (11), B-cell lymphoma (BCL), lung cancer (LC), hepatocellular carcinoma (HCC) (10), clear cell renal cell carcinoma (CCRCC) (12), colorectal cancer (CRC) (13), and pancreatic adenocarcinoma (PAAD) (14–18), but is not expressed in most normal tissues (10, 19, 20). High expression of MYOF has been associated with shorter survival in patients with breast (21), pancreatic (16, 17, 22), and colorectal (13, 23) cancers. MYOF has been found to be highly expressed in lipogenic pancreatic cancer cells, and to be involved in maintaining high oxidative phosphorylation (OXPHOS) activity and mitochondrial network structure (22). The depletion of MYOF from lipogenic pancreatic cancer cells was found to inhibit ATP production and trigger autophagy, and MYOF has been reported to affect intracellular levels of reactive oxygen species (ROS) (22), Although MYOF was found to regulate *EGFR* and its downstream epithelial mesenchymal transition (EMT) in nasopharyngeal carcinoma (24), suggesting that MYOF ameliorates metastasis of several types of cancer, the complex processes by which MYOF regulates the migration of gastric cancer cells remain incompletely understood.

To determine whether *MYOF* expression is a biomarker for gastric cancer diagnosis and prognosis, the present study comprehensively analyzed the expression of *MYOF* and its co-expressed factors in gastric cancer and the association between *MYOF* expression and prognosis in patients with gastric cancer using publicly available databases. In addition, the effects of myoferlin on intracellular ROS levels and cell migration were assessed *in vitro* in gastric cancer cells.

## Materials and methods

### Cell culture

The gastric cancer cell lines HGC27, SNU1 and NCI-N87 were purchased from the American Type Culture Collection (ATCC), MKN45 cells were purchased from the Chinses Academy of Science and cultured in Dulbecco’s modified Eagle’s medium (DMEM) (Gibco) supplemented with 10% fetal bovine serum (FBS) (Invitrogen), 100 U/mL penicillin G and 100 μg/mL streptomycin sulfate (Beyotime) at 37°C in an atmosphere containing 5%CO_2_. Cell identity was authenticated by short tandem repeat (STR) profiling and the cultures cells were tested monthly in our laboratory for mycoplasma using MycoSEQ Detection Kits (Applied Biosystems). Gastric cancer cells between the third and sixth passage were utilized for *in vitro* experiments.

### *MYOF* knockdown and overexpression

*MYOF* was knocked down in NCI-N87 and MKN45 cells using lentiviral shRNA vectors (GenePharma Technology) designed to target the sequences 5′-GAAAGAGCTGTGCATTATAAA-3’ and 5′-GCTGTGGAGAAGAAGTTTAAC-3′ in the *MYOF* coding region. Stable knockdown cells were isolated by selecting for puromycin resistance.

To overexpress *MYOF*, human *MYOF* complementary DNA (cDNA) was amplified and inserted into a lentiviral vector; as a control, an empty lentiviral vector (GenePharma Technology) was prepared similarly. The vectors were introduced into HGC27 and SNU1 cells, and cells were selected for puromycin resistance 2 weeks before experiments performed as previously described (25).

### Quantitative real-time PCR

qRT-PCR was performed as using primers for human *MYOF* (forward, 5’-CATTGACTTGGTGATCGGCTAT-3’; reverse, 5’-CCTGACTGCATGTCCAACC-3’); human *Twist1* (forward, 5’-GTCCGCAGTCTTACGAGGAG-3’; reverse, 5’- GCTTGAGGGTCTGAATCTTGCT-3’); human *Arpc3* (forward, 5’-GTGCAATTCCAAAAGCCAAGG-3’; reverse, 5’-GGCTCTCATCACTTCATCTTCC-3’); and human *GAPDH* (forward, 5’-GGAGCGAGATCCCTCCAAAAT-3’; reverse, 5’-GGCTGTTGTCATACTTCTCATGG-3’), as previously described (26).

## Database analysis

### Data acquisition

Data on gene expression in normal and tumor tissues of gastric cancer patients were downloaded from the TCGA and GEO (GSE27342) databases and analyzed using the UALCAN web tool (27).

### Immunohistochemistry

Immunohistochemical analysis was performed using a two-step procedure on a Dako REAL™ Envision™ Detection System (Agilent Technology), according to the manufacturer’s instructions. Briefly, following antigen retrieval, tissue sections were incubated with primary anti-MYOF antibody (Sigma, HPA014245, 1:300) at 4°C overnight, followed by incubation with horseradish peroxidase (HRP) labeled secondary antibodies at 37°C for 30 minutes, with staining visualized by incubating the samples with diaminobenzidine.

### Kaplan–Meier plotter

The prognostic value of *MYOF* expression in gastric cancer patients was evaluated using the GEO database (28), the database included GSE15459, GSE14210, GSE29272, GSE22377, GSE51105 and GSE62254 data sets (29). Hazard ratios (HRs), 95% confidence intervals (CIs) and log rank p-values were calculated.

### Analysis of factors correlating with *MYOF*

Genes correlating with *MYOF* expression were evaluated using the cBioPortal, MEM and GEPIA analysis tools (30, 31). The overlapping region on the Venn diagram of the three datasets was determined, and the relationship between *MYOF* expression and correlating factors was evaluated using Spearman correlation analysis. The MEM dataset was subsequently uploaded into the Database for Annotation, Visualization, and Integrated Discovery (DAVID) web tool (32). GO function enrichment analysis was utilized, with a P value <0.05 defined as the cutoff criterion.

### cBioPortal analysis of gastric cancer data in the Cancer Genomics database

*MYOF* and correlating factors were analyzed using the cBioPortal tool. The primary search terms included alterations (mutations, amplifications, high mRNA levels, extensive deletions, and multiple alterations) and predicted locations of the alterations, with the default settings across samples curated from gastric cancer.

### Gene set enrichment analysis

The biological function of *MYOF* gene was analyzed by GSEA (33). Annotated gene sets c2.cp.kegg.v5.2.symbols.gmt were selected as the reference gene sets. The level of *MYOF* expression was set as a phenotype label. Pathways enriched in each phenotype were identified based on false defection rate q values <0.05 and a normalized enrichment scores (NSE) >1.

### GSH/GSSG measurements

Concentrations of GSH and GSSG were measured using a GSH and GSSG Assay Kit (Beyotime Biotechnology, China, S0053) according to manufacturer’s instructions. Briefly, 1×10^6^ cells cultured in each well of a six-well plate were treated with NAC or vehicle for 24 hours, washed with PBS and centrifuged twice. The protein removal reagent M solution was added to each cell sample and the cells were subjected to two execute quick freeze-thaw cycles using liquid nitrogen and a 37°C water bath. The cells were kept at 4°C for 5 minutes and centrifuged at 10,000×*g* for 10 minutes. Each supernatant of cell samples was added to a well on a microtiter plate, and the absorbance of each well was measured at a wavelength of 410 nm on a microplate reader. GSH and GSSG in the cell samples were measured by colorimetric method, and the GSH/GSSG ratio of each sample was calculated.

### NADPH/NADP+ measurements

Intracellular NADPH and NADP+ concentrations were measured using NADP+/NADPH (WST-8) assay kits (Beyotime Biotechnology, China, S0179), according to the manufacturer’s instructions. Briefly, 1×10^6^ cells cultured in each well of a six-well plate were treated with NAC or vehicle for 24 hours. The cells were lysed by adding 400 μl of extraction buffer, and the preparations were centrifuged at 12,000×*g* for 10 minutes at 4°C. The supernatants were collected and kept on ice in the dark. Total NADP (i.e., NADP^+^ plus NADPH) was determined by incubating 50 μl of each supernatant with 100 μl G6PDH working solution for 10 minutes at 37°C and measuring absorbance at 450 nm on a microplate reader. NADPH was determined by incubating the supernatant in a water bath at 60°C for 30 minutes, followed by incubating 50 μl of each sample with 100 μl G6PDH working solution for 10 minutes at 37°C and measuring absorbance at 450 nm on a microplate reader. Concentrations of total NADP and NADPH were assessed by comparison with standard curves, and NADP^+^ concentrations were determined by subtracting NADPH from total NADP concentrations, with [NADPH]/[NADP^+^] ratios calculated as [NADPH]/([NADP_total_] - [NADPH]).

### ROS measurements

Intracellular ROS levels were measured using Reactive Oxygen Species Assay Kits (Beyotime Biotechnology, China, S0033S). Briefly, cells seeded in six-well plates were incubated with 5 μM N-acetyl-L-cysteine (NAC) (Selleck, S1623) or vehicle for 24 hours at 37°C. The cells were washed with PBS, trypsinized, neutralized and washed again, followed by incubation with DCFH-DA for 30 minutes at 37°C and three washes with PBS. Intracellular ROS was assessed by flow cytometry.

### Migration assays

Migration assays were performed using transwell filter chambers (Merck), according to the manufacturer’s instructions. Briefly, 1×10^5^ cells in 100 μl of serum-free medium were seeded into each upper compartment of a 24-well plate, and DMEM containing 10% FBS was added to each lower compartment. The chambers were placed onto the lower compartments and incubated at 37°C for 24 hours. The chambers were washed with PBS, and the cells were fixed in 1% paraformaldehyde and stained with 1% crystal violet. Cells that had migrated through the membranes were quantified. Three random microscopic fields of each membrane were selected and the numbers of cells averaged. Each experiment was repeated at least three times independently, as previously described (34).

### Immunofluorescence

NCI-N87 cells were cultured on eight-well plates (Millipore) for 48 hours, washed twice with PBS, fixed in 4% paraformaldehyde for 20 minutes at room temperature, and permeabilized with 0.1% Triton X-100 for 20 minutes. The cells were incubated with 5% normal goat serum for 15 minutes at room temperature, followed by incubation with primary anti-MYOF antibody (Sigma, HPA014245; 1:300 in PBS) overnight at 4°C. The cells were washed for three times with PBS and incubated with species-specific secondary antibody at room temperature for 30 minutes. Nuclei were subsequent stained with DAPI, and the cytoskeleton was stained with phalloidin. The cells were observed and recorded using a Carl Zeiss microscope and ZEN software (ZEISS Company).

### Western blotting

Cells were lysed in RIPA buffer containing 1× protease inhibitor cocktail, and protein concentrations were measured using a BCA protein assay kit (Beyotime Biotechnology, P0012S). Aliquots containing 30 μg of protein were resolved on 10% SDS-PAGE gels and the proteins transferred to PVDF membranes (Beyotime Biotechnology, FFP24). The membranes were incubated with 5% bovine serum albumin (BSA) (Beyotime Biotechnology, ST025-5g) in TBST to block nonspecific binding. The membranes were subsequently incubated with primary anti-MYOF (Sigma, HPA014245; diluted 1:1000 in TBST) and anti-GAPDH (Cell Signaling Technology, #5174; diluted 1:1000 in TBST) antibodies overnight at 4°C, followed by incubation with species-specific secondary antibodies for 2 hours at room temperature. Binding was visualized using the ECL substrate solution (Beyotime Biotechnology, P0018FS) and imaged using an infrared imaging system.

### Ethics statement

This study was approved by the Ethics Committee of Tai’an City Central Hospital, and all experiments conformed to the ethical principles of the World Medical Association Declaration of Helsinki.

### Statistical analysis

All results were reported as mean± standard deviation (S.D.). Quantitative data were compared in two groups using independent sample *t*-tests and in more than two groups by one-way analysis of variance (ANOVA). Non-parametric data were compared in two groups using two-tailed Mann–Whitney U-tests and in more than two groups using Kruskal–Wallis tests in combination with Dunn’s multiple comparison post-tests. All statistical analyses were performed using R software for windows (cran.r-project.org), with two-tailed *P*-values <0.05 considered statistically significant.

## Results

### Expression of *MYOF* and correlated genes in gastric cancer tissues

**Figure 1:**
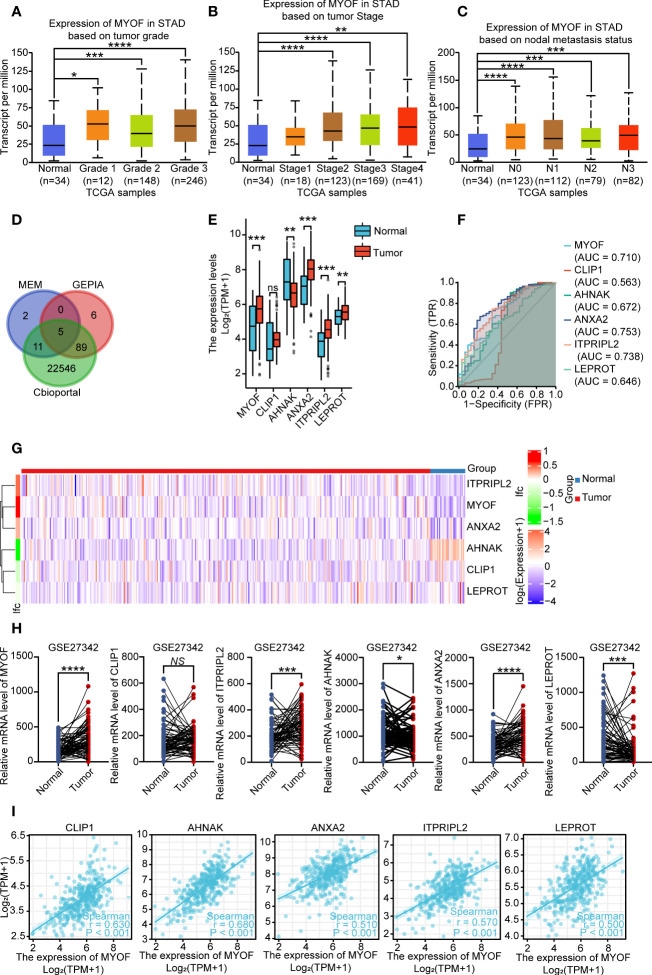
Expression of *MYOF* mRNA and correlated genes in gastric cancer and normal gastric tissues. **(A)** *MYOF* mRNA levels in gastric cancer samples from the TCGA database according to tumor grade, with Grade 1 indicating well-differentiated (low grade) tumors, Grade 2 indicating moderately differentiated (intermediate grade) tumors and Grade 3 indicating poorly differentiated (high grade) tumors **(B)** *MYOF* mRNA levels in gastric cancer samples with different AJCC stages from the TCGA databases. **(C)** *MYOF* mRNA levels in gastric cancer samples from the TCGA database differing in lymph node metastasis status, with N0 indicating no regional lymph node metastases and N1, N2, and N3 indicating metastases in 1–3, 4–9 and ≥10 axillary lymph nodes. **(D)** Venn diagram showing factors correlating with *MYOF* expression identified with the cBioPortal, GEPIA, and MEM web tools. The shared genes were found to be *CLIP1*, *AHNAK*, *ANXA2*, *ITPRIPL2* and *LEPROT*. **(E)** Expression of *MYOF*, *CLIP1*, *AHNAK*, *ANXA2*, *ITPRIPL2* and *LEPROT* mRNAs in gastric cancer and normal gastric tissues in the TCGA database. **(F)** Receiver operating characteristic (ROC) curves comparing the ability of *MYOF*, *CLIP1*, *AHNAK*, *ANXA2*, *ITPRIPL2* or *LEPROT* mRNA levels to predict gastric cancer. FPR: false positive rate; TPR: true positive rate. **(G)** Heatmap representation of *MYOF*, *CLIP1*, *AHNAK*, *ANXA2*, *ITPRIPL2* and *LEPROT* expression profiles in tumor and normal tissues from the TCGA-STAD database. Genes with higher and lower expression in tumor samples are shown in red and blue, respectively. **(H)** Validation of *MYOF*, *CLIP1*, *AHNAK*, *ANXA2*, *ITPRIPL2* and *LEPROT* mRNA expression levels in gastric cancer and normal gastric tissues in the GEO database (GSE27342). **(I)** Correlations between *MYOF* mRNA levels and the expression of correlating factors in human gastric cancer tissues from the TCGA-STAD database. Data are expressed as mean ± S.D., and *P* values were calculated using unpaired two-tailed Student’s *t*-tests or ANOVA, as appropriate. *NS*, not significant, *P<0.05, **P<0.01, ***P<0.001, ****P<0.0001.

To explore the function of *MYOF* in gastric cancer, the expression of *MYOF* mRNA was evaluated in the TCGA-STAD and GEO databases. Compared with normal gastric tissue, the levels of *MYOF* mRNA were significantly higher in Grades 1 (P<0.05), 2 (P<0.001) and 3 (P<0.0001) gastric cancer tissue, with *MYOF* mRNA levels increasing with increasing grade of gastric cancer (**Figure 1A**). Although *MYOF* mRNA expression in AJCC stage 1 gastric cancer tissues was no higher than that in normal gastric tissues (P>0.05), the levels of *MYOF* mRNA were significantly higher in Stages 2 (P<0.0001), 3 (P<0.0001) and 4 (P<0.01) gastric cancer than in normal gastric tissue (**Figure 1B**). Moreover, *MYOF* expression was significantly higher in tumor tissue from patients with N0 (P<0.0001), N1 (P<0.0001), N2 (P<0.001) and N3 (P<0.001) gastric cancer than in normal gastric tissue (**Figure 1C**).

Application of the cBioPortal, MEM and GEPIA web tools to identify genes that correlate with *MYOF* found that the expression of five shared genes in the three datasets correlated with *MYOF* mRNA expression. These included *CLIP1*, which encodes CAP-gly-domain containing linker protein 1, a protein that links endocytic vesicles to microtubules; *AHNAK*, which encodes the nucleoprotein AHNAK, a protein involved in blood–brain barrier formation, cell structure and migration, cardiac calcium channel regulation, and tumor metastasis; *ANXA2*, which encodes annexin A2, a protein involved in regulating cellular growth and signal transduction pathways and correlating with resistance to treatment of several types of cancer; *ITPRIPL2*, which encodes inositol 1,4,5-trisphosphate receptor interacting protein like 2, a protein that can enhance the sensitivity of inositol 1,4,5-trisphosphate receptor (ITPR) to intracellular calcium signaling; and *LEPROT*, which encodes leptin receptor overlapping transcript, which is involved in cell surface expression of growth hormone and leptin receptors and alters receptor-mediated cell signaling (**Figure 1D**).

Evaluation of the expression of *MYOF* and these correlating genes in gastric cancer and normal gastric tissues in the TCGA-STAD database showed that the levels of *MYOF* (P<0.001), *ANXA2* (P<0.01), *LEPROT* (P<0.01) and *ITPRIPL2* (P<0.001) mRNAs were significantly higher, and the levels of *AHNAK* mRNA significantly lower (P<0.01) in gastric cancer than in normal gastric tissue samples, but that there was no difference in *CLIP1* mRNA levels (P>0.05; **Figure 1E**). ROC curve analysis evaluating the predict value of expression of *MYOF* and the other five genes showed that high expression of *MYOF* was more likely to be found in tumor than in normal tissues (cut-off, 4.838; AUC, 0.710; 95% CI, 0.614–0.806; Youden index, 0.562; sensitivity, 0.765; specificity, 0.562) (**Figure 1F**; **Table 1**). DeLong’s tests showed that the efficiency of *MYOF* was significantly better than that of *CLIP1* (P < 0.001) and *LEPROT* (P = 0.037), and a heatmap showed distinct differences in gene expression profiles between gastric cancer and normal gastric tissue (**Figure 1G**). The expression of these genes in gastric cancer tissues was further evaluated in the GEO database (GSE 27342), which found that the levels of *MYOF* (P<0.0001), *ANXA2* (P<0.0001) and *ITPRIPL2* (P<0.001) mRNAs were significantly higher, and the levels of *LEPROT* (P<0.001) and *AHNAK* (P<0.05) mRNAs significantly lower, in gastric cancer than in normal gastric tissue samples, with no significant difference in *CLIP1* mRNA expression (P>0.05; **Figure 1H**). Evaluation of the relationship between *MYOF* mRNA expression and the expression of the five correlating genes in the TCGA-STAD database showed that *MYOF* mRNA levels were positively associated with *CLIP1* (R=0.630, P<0.0001), *AHNAK* (R=0.680, P<0.001), *ANXA2* (R=0.510, P<0.001), *ITPRIPL2* (R=0.570, P<0.001), and *LEPROT* (R=0.500, P<0.001) mRNAs (**Figure 1I**).

**Table 1:** Tumor predicted values of *MYOF* and correlating genes.

Gene	AUC	CI	Cut-off value	Sensitivity	Specificity
*MYOF*	0.71	0.614-0.806	4.838	0.765	0.562
*CLIP1*	0.563	0.412-0.715	3.231	0.891	0.469
*AHNAK*	0.672	0.555-0.788	7.882	0.904	0.469
*ANXA2*	0.753	0.653-0.854	7.651	0.675	0.781
*ITPRIPL2*	0.738	0.647-0.829	4.063	0.747	0.656
*LEPROT*	0.646	0.550-0.743	5.539	0.523	0.719

### Prognostic value of *MYOF* mRNA level in gastric cancer patients

**Figure 2:**
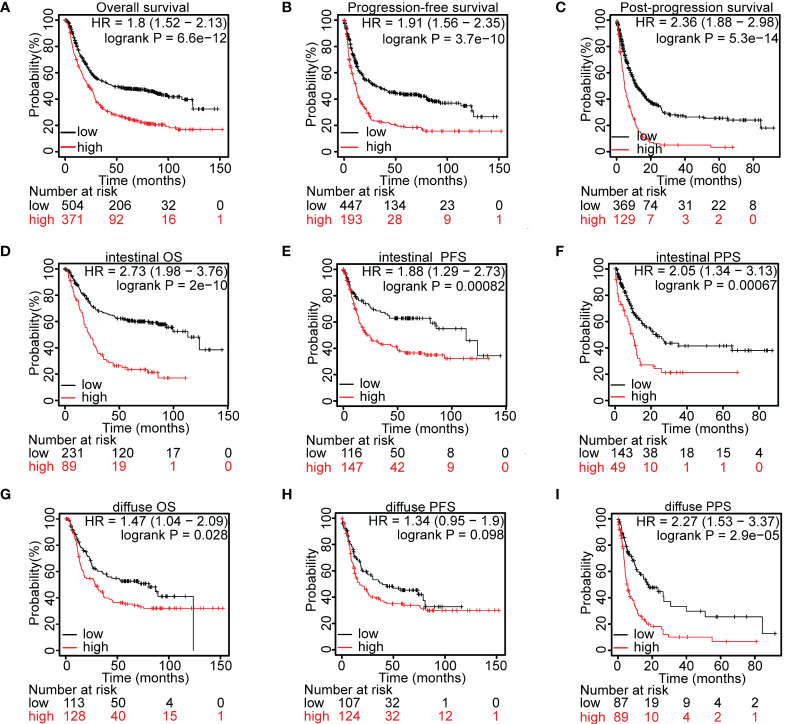
Prognostic value of *MYOF* mRNA level in gastric cancer patients. **(A–C)** Kaplan–Meier analyses of **(A)** overall survival (OS), **B)** progression-free survival (PFS), and **(C)** post-progression survival (PPS) of gastric cancer patients with high and low levels of *MYOF* mRNA expression in the GEO database. **(D–F)** Kaplan–Meier analyses of **(D)** OS, **(E)** PFS, and **(F)** PPS of patients with intestinal type gastric cancer and high and low levels of *MYOF* mRNA expression from the GEO database. **(G–I)** Kaplan–Meier analyses of **(G)** OS, **(H)** PFS, and **(I)** PPS of patients with diffuse type gastric cancer and high and low levels of *MYOF* mRNA expression from the GEO database. Survival times were compared between groups using the Mantel–Cox test.

Kaplan–Meier analysis of gastric cancer patients in the GEO database showed that high expression of *MYOF* was significantly associated with poorer overall survival (OS; HR = 1.80, 95% CI 1.52–2.13, P<0.0001; **Figure 2A**), progression-free survival (PFS; HR = 1.91; 95% CI 1.56–2.35, P<0.0001; **Figure 2B**) and post-progression survival (PPS; HR = 2.36, 95% CI 1.88–2.98, P<0.0001; **Figure 2C**). The Lauren classification method has been used to sort patients based on various distinct clinical and molecular characteristics, including histology, epidemiology, etiology, biological behavior, and prognosis (35). Patients with two major histological subtypes of gastric cancer, intestinal type and diffuse type, were therefore analyzed. Compared with low *MYOF* expression, high *MYOF* expression in patients with intestinal type gastric cancer was associated with significantly poorer OS (HR = 2.73, 95% CI 1.98–3.76, P<0.0001; **Figure 2D**), PFS (HR = 1.88, 95% CI 1.29–2.73, P<0.001; **Figure 2E**) and PPS (HR = 2.05, 95% CI 1.34–3.13, P<0.001; **Figure 2F**). Among patients with diffuse type cancer, high *MYOF* expression was associated with poorer OS (HR = 1.47, 95% CI 1.04–2.09, P=0.028; **Figure 2G**), and PPS (HR = 2.27, 95% CI 1.53–3.37, P<0.0001; **Figure 2H**), but not PFS (HR = 1.34, 95% CI 0.95–1.9, P=0.098; **Figure 2I**) compared with low *MYOF* expression. Taken together, these findings indicated that high expression of *MYOF* was associated with shorter survival in patients with gastric cancer.

### cBioPortal analysis of alterations in *MYOF* and correlated genes

**Figure 3:**
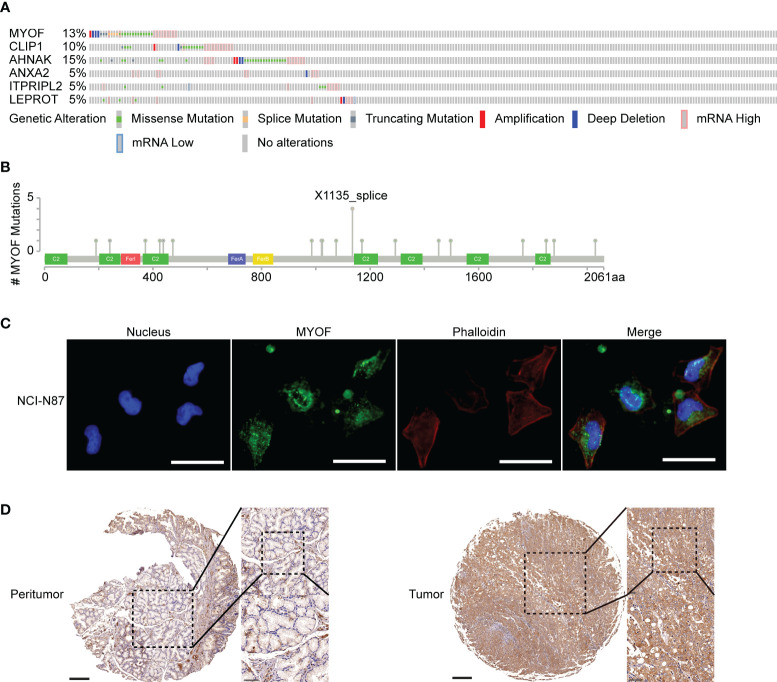
Alterations in the *MYOF* and correlating genes using the cBioPortal webtool. **(A)** Genetic alterations in the *MYOF*, *CLIP1*, *AHNAK*, *ANXA2*, *ITPRIPL2* and *LEPROT* genes of patients in the TCGA-STAD cohort derived from the cBioPortal website. **(B)** Graphic representation of the location, frequency, and mutation hotspots of the *MYOF* gene in gastric cancer patients from the TCGA cBioPortal. **(C)** Immunofluorescence staining of NCI-N87 cells for MYOF (MYOF, green; DAPI, blue; Palloidin, red). Scale bar, 50 μm, 400× magnification. **(D)** Representative images from gastric cancer tissue and peritumor sections stained with anti-MYOF antibody. Scale bars, 200 μm, 50× magnification (main images; left); 50 μm, 200× magnification (magnified view of the regions in the dotted boxes; upper right), and 50 μm, 400× magnification (magnified view of the regions in the dotted boxes; lower right).

The *MYOF*, *CLIP1*, *AHNAK*, *ANXA2*, *ITPRIPL2* and *LEPROT* genes in tissues of patients with gastric cancer were found to be altered by amplification, extensive deletion, truncating mutations, splice mutations, missense mutations, and high/low mRNA expression (**Figure 3A**). Use of the cBioPortal web tool showed that 13%, 10%, 15%, 5%, 5% and 5% of the *MYOF*, *CLIP1*, *AHNAK*, *ANXA2*, *ITPRIPL2* and *LEPROT* genes, respectively, were altered in these tissue samples. *MYOF* missense mutations were the most common type of gene alteration (7.36%), with these other types of gene alterations also observed (**Figure 3A**). Structurally, MYOF protein was characterized by multiple functional C2 domains, which have been reported to correlate with the proliferative and metastatic behaviors of cancers (8, 11), as well as small conserved 60–70 residue Ferlin-specific sequences (FerA, FerB and Ferl domains). In addition, splice mutations at one hot spot (X1135) were observed in samples from four patients (**Figure 3B**).

### Localization of MYOF protein in cells and tumor tissues

MYOF protein was shown to be located at the plasma membrane, at which it functions to repair the lipid bilayer, especially in skeletal muscle cells, of tissues exposed to heightened mechanical stress (36). In pancreatic cancer cells, MYOF protein was found to localize at lysosome membranes (16). To determine the location of MYOF in gastric cancer cells, immunofluorescence assays were performed using NCI-N87 cells, which express high levels of MYOF. Most MYOF protein was found to localize to the cytoplasm, visible as bright patches representing aggregates of MYOF (**Figure 3C**). To further confirm the subcellular location of MYOF in gastric cancer cells, an immunohistochemical analysis was performed using a commercial microarray of gastric cancer tissues (TMA). In agreement with the immunofluorescence results, MYOF protein was found to be upregulated in tumor tissue, mainly within the cytoplasm, whereas little or no MYOF protein was detected in gastric glandular cells (**Figure 3D**).

### *MYOF* may play multiple roles in gastric cancer

**Figure 4:**
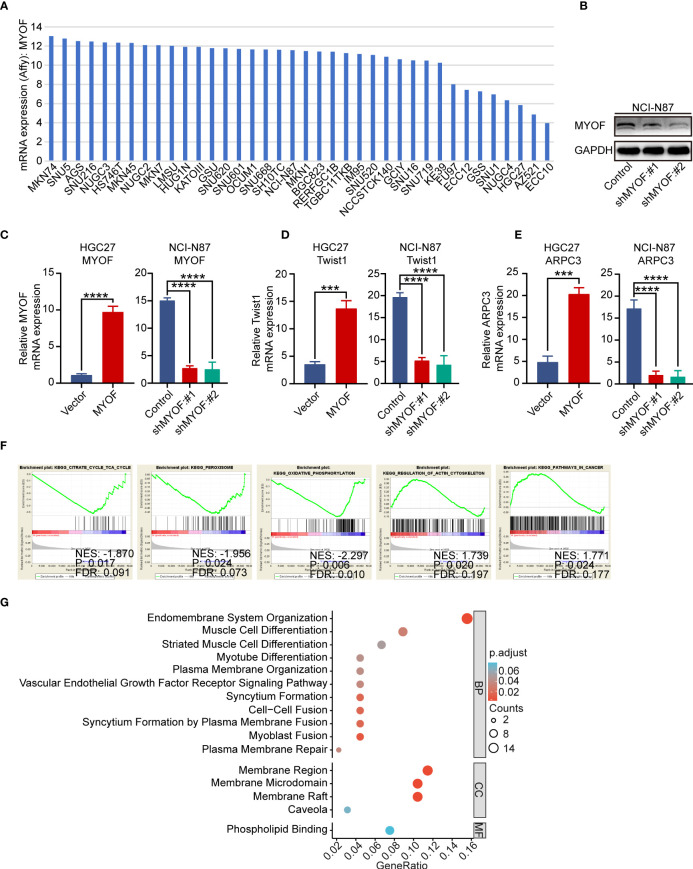
Biological function of *MYOF* in gastric cancer cells. **(A)** *MYOF* mRNA expression in gastric cancer cell lines from the Cancer Cell Line Encyclopedia database. **(B)** Effect of MYOF knockout on the expression of MYOF protein in NCI-N87 cells infected with empty lentiviral vector (Control) and with vectors expressing sh*MYOF* #1, and #2, as determined by western blotting. GAPDH was used as the protein loading control. **(C)** Effect of MYOF knockout on *MYOF* mRNA expression in indicated cells by qRT-PCR. **(D, E)** Effects of MYOF knockout or overexpression on the expression of **(D)** *Twist1* and **(E)** *ARPC3* mRNAs, as determined by qRT-PCR. The data shown are the mean ± SD relative mRNA expression from three independent experiments, using triplicates of each sample in each experiment. *P* values were determined using unpaired two-tailed Student’s *t*-tests or ANOVA. ***P<0.001, ****P<0.0001. **(F)** Gene Set Enrichment Analysis of identified KEGG pathways in gastric cancer tissues with high and low *MYOF* expression levels. **(G)** Representative Gene Ontology (GO) pathways associated with biological processes (BP), cellular components (CC) and molecular functions (MF).

The expression of *MYOF* in gastric cancer cells was further investigated using the Cancer Cell Line Encyclopedia (CCLE) database. *MYOF* was found to be highly expressed in most gastric cancer cells (**Figure 4A**), and to be over-expressed in HGC27 and SNU1 cells (P<0.0001 each). Knockdown of *MYOF* in NCI-N87 and MKN45 cells using lentiviral shRNA vectors sh*MYOF*#1 and sh*MYOF*#2 significantly silenced the expression of *MYOF* in NCI-N87 and MKN45 cells (P<0.0001 each; **Figures 4B, C**; **Supplementary Figures 1A, B**). PCR analysis showed that lentiviral vector knockdown of *MYOF* in NCI-N87 and MKN45 cells suppressed the expression of *Twist1* and *Arpc3* mRNAs, which encode markers of cell motility (P<0.0001 each), whereas infection with vectors overexpressing *MYOF* enhanced the expression of *Twist1* and *Arpc3* mRNAs (P<0.001 each) (**Figures 4D, E**; **Supplementary Figures 1C, D**). Taken together, these findings suggested that *Twist1* and *Arpc3* mRNAs are positively regulated by *MYOF* and that *MYOF* might promote the motility of gastric cancer cells.

To further characterize the *MYOF*-associated signaling pathways, GSEA was performed to enrich the KEGG pathways in groups of patients in the TCGA-STAD database with high and low expression of *MYOF*. The cutoff criteria were defined as a false discovery rate (FDR) <0.05 and an absolute value of the enrichment score (ES) >0.5. These analyses showed that the meaningful signaling pathways included “KEGG_CITRATE_CYCLE_TCA_CYCLE”, “KEGG_PEROXISOME”, “KEGG_OXIDATIVE PHOSPORYLATION”, “KEGG_REGULATION_OF_ACTIN_CYTOSKELETON” and “KEGG_PATHWAYS_IN_CANCER” (**Figure 4F**). Gene Ontology (GO) analysis to determine the functional characteristics of *MYOF* and correlating genes in the MEM database showed significant enrichment of the GO functions “endomembrane system organization”, “muscle cell differentiation”, “striated muscle cell differentiation”, “myotube differentiation”, “plasma membrane organization”, “vascular endothelial growth factor receptor signaling pathway”, “syncytium formation”, “cell-cell fusion”. “syncytium formation by plasma membrane fusion”, “myoblast fusion”, “plasma membrane repair”, “membrane region”, “membrane microdomain” and “membrane raft” (all P<0.05; **Figure 4G**). Because almost all of these signaling pathways and GO terms have been reported to participate in tumor progression, these results suggested the extensive involvement of MYOF in gastric cancer progression.

### *MYOF* induced intracellular ROS enrichment is responsible for regulating gastric cancer cell migration

**Figure 5:**
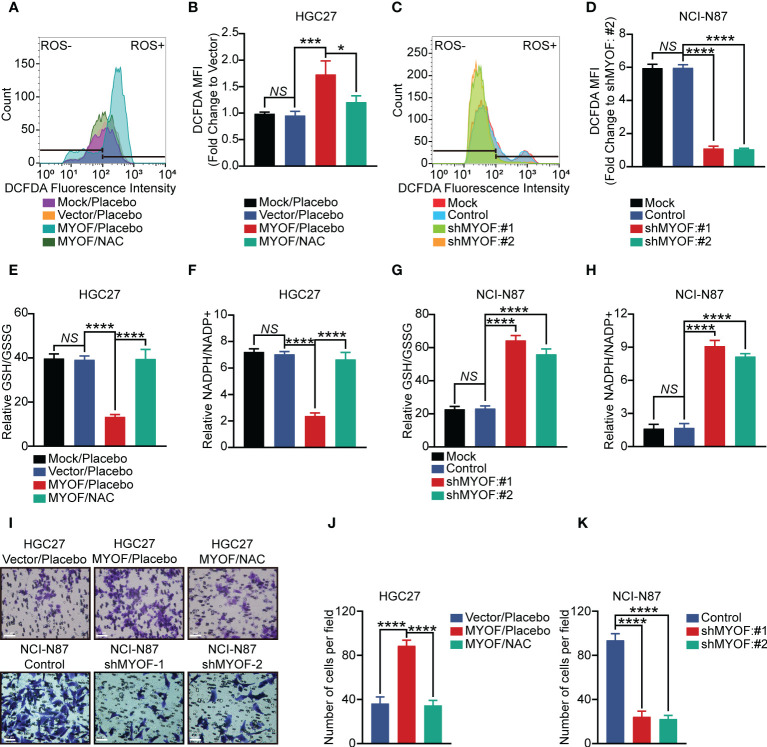
MYOF promotion of gastric cancer cell motility and intracellular reactive oxygen species (ROS). **(A, B)** Intracellular ROS levels **(A)** and DCFDA geometric mean fluorescence intensity (MFI) **(B)** of HGC27 gastric cancer cells alone (wild-type) or infected with empty vector or vector encoding *MYOF*, with the latter treated with vehicle or NAC (5 μM) for 24 **h** The data in **(B)** are shown as the mean ± SD fold changes in MFI compared with HGC27-Vector cells from three independent experiments. **(C, D)** Intracellular ROS levels **(C)** and DCFDA geometric mean fluorescence intensity (MFI) **(D)** of NCI-N87 gastric cancer cells alone (wild-type) or infected with empty vector or vector encoding sh*MYOF*#1 or sh*MYOF*#2. The data in **(D)** are shown as the mean ± SD fold changes in MFI compared with NCI-N87 sh*MYOF*#2 cells from three independent experiments. **(E, F)** Intracellular GSH and GSSG levels, expressed as GSH/GSSG ratios **(E)**, and intracellular NADPH and NADP+ levels, expressed as NADPH/NADP+ ratios **(F)**, of HGC27 gastric cancer cells alone (wild-type) or infected with empty vector or vector encoding *MYOF*, with the latter treated with vehicle or NAC (5 μM) for 24 **h** Results are expressed as the mean ± SD of three independent experiments. **(G, H)** Intracellular GSH and GSSG levels, expressed as GSH/GSSG ratios **(G)**, and intracellular NADPH and NADP+ levels, expressed as NADPH/NADP+ ratios **(H)**, of NCI-N87 gastric cancer cells alone (wild-type) or infected with empty vector or vector encoding sh*MYOF*#1 or sh*MYOF*#2. Results are expressed as the mean ± SD of three independent experiments. **(I)** Representative images of cell migration assays. Scale bar, 20 μm, 200× magnification. **(J, K)** Statistical analysis of migratory cells. Results are reported as the mean ± SD of three independent experiments, using triplicates of each sample in each experiment. *P* values were determined using unpaired two-tailed Student’s *t*-tests or ANOVA. *NS*, not significant, *P<0.05, ***P<0.001, ****P<0.0001.

“KEGG_PEROXISOME” and “KEGG_OXIDATIVE PHOSPHORYLATION” were found to be among the KEGG pathways most significantly altered in gastric cancer. Moreover, ROS has been shown to play a role in the activation of proto-oncogenes and to act as signaling factor to induce cancer cell growth and metastasis (37). We hypothesized that MYOF could regulate gastric cancer cell migration by altering ROS levels. Intracellular ROS levels were found to be higher in MYOF-overexpressing than in control HGC27 (P<0.001) and SNU1 (P<0.0001) gastric cancer cells (**Figures 5A, B**; **Supplementary Figures 2A, B**). Similarly, knockdown of *MYOF* with sh*MYOF*#1 and sh*MYOF*#2 reduced intracellular ROS levels in NCI-N87 and MKN45 cells (P<0.0001 each; **Figures 5C, D**; **Supplementary Figures 2C, D**). Furthermore, addition of N-acetyl-L-cysteine (NAC), a potent inhibitor of ROS, significantly inhibited the MYOF-induced production of intracellular ROS in HGC27 (P<0.05) and SNU1 (P<0.0001) cells (**Figures 5A, B**; **Supplementary Figures 2A, B**).

Glutathione was a major ROS scavenger in cancer cells, being present in thiol reduced (GSH) and oxidized (GSSG) states, with GSH being predominant. NADPH was a crucial cofactor and electron donor that replenishes GSH levels and maintains redox balance (38). MYOF was found to alter the amounts of GSH and GSSG in gastric cancer cells, reducing the GSH/GSSG ratio in HGC27 and SNU1 cells (P<0.0001; **Figures 5E**; **Supplementary Figures 2E**). Moreover, treatment with NAC effectively increased the GSH/GSSG ratio in MYOF-overexpressing HGC27 and SNU1 gastric cancer cells, (P<0.0001 each). MYOF also reduced the NADPH/NADP+ ratio in MYOF-overexpressing HGC27 and SNU1 (P<0.0001 each), with the addition of NAC significantly increasing the NADPH/NADP+ ratio in these cells (P<0.0001 each; **Figure 5F**; **Supplementary Figures 2F**). Intriguingly, knockdown of MYOF significantly increased the GSH/GSSG and NADPH/NADP+ ratios in NCI-N87 and MKN45 cells (P<0.0001 each; **Figures 5G, H**; **Supplementary Figures 2G, H**). Infection of gastric cancer cells with empty vector did not alter intracellular ROS levels, GSH/GSSG ratios and NAPDH/NAPD+ ratios (P>0.05 each; **Figures 5A–H**; **Supplementary Figures 2A–H**).

The ability of MYOF to enhance ROS in gastric cancer cells suggested that alterations in ROS levels could alter the migratory ability of these cells. Transwell assays showed that MYOF increased the migration ability of HGC27 and SNU1 cells (P<0.0001 each), whereas the addition of NAC could attenuate the MYOF-associated cell migration (P<0.0001). Furthermore, knockdown of MYOF significantly alleviated the migration ability of NCI-N87 and MKN45 cells (P<0.0001 each; **Figures 5I–K**; **Supplementary Figures 2I–K**).

## Discussion

Because MYOF had been shown to be involved in sustaining enhanced lysosome function in pancreatic cancer by defending against membrane stressors (16), previous studies focused on the role of MYOF in the regulation of intracellular signaling and mitochondrial metabolism in cancer cells. Little was known, however, about the function of MYOF in regulating intracellular ROS production in cancer cells. The present study found that *MYOF* is broadly upregulated in gastric cancer cell lines and patients’ tumor specimens, and that high expression of *MYOF* could predict relatively poor prognosis in patients with gastric cancer. Moreover, MYOF was found to ameliorate the migratory ability of gastric cancer cells and intracellular ROS levels, whereas the antioxidant NAC could inhibit MYOF-associated cell migration. Taken together, these findings suggest that myoferlin is a promising prognostic biomarker for gastric cancer, and plays a role in regulating redox equilibrium and migration.

Ferlins were a family of multiple C2 domain proteins that are involved in vesicle fusion and membrane trafficking. A typical C2 domain consisted of a beta-sandwich, composed of eight beta-strands with coordinating calcium ions that participate in binding phospholipids. Several C2 domains did not bind calcium but still bind membranes (6). C2 domains are present in many proteins, with most C2 domains participating in membrane functions, including vesicular transport (synaptotagmin), GTPase regulation (Ras GTPase activating protein) and lipid modification (phospholipase C).

Ferlins differ in tissue and developmental specificity, with animal models containing defects in ferlins having pathologic characteristics resulting from defects in vesicle fusion. Because ferlins are involved in vesicle trafficking, they interact with cytoskeletal motors, other vesicle-associated trafficking proteins and transmembrane receptors or channels, such as PINCH-1, EGFR, VEGFA, IGFR-1, VEGFR-2, Dynamin-2 (endothelial cells) and EHD2 (myoblasts). Dysferlin and myoferlin have crucial functions in cellular trafficking, with both being involved in trafficking and recycling of IGFR-1, suggesting that myoferlin is involved in modulating muscle growth (36).

Myoferlin was found to be upregulated in proliferating mononuclear cells, becoming down-regulated with myogenic maturation. Moreover, myoferlin was found to be necessary for myoblast fusion during the process of muscle development and regeneration. Absence of myoferlin may lead to delayed transferrin recycling in myoblasts, and knockdown of myoferlin could inhibit clathrin- and caveolin-dependent endocytosis in COS7 cells (39). The C2B domain of myoferlin may interact with EHD2 *via* an asparagine-proline-phenylalanine (NPF) motif, indirectly modulating the disassembly or reorganization of the cytoskeleton that accompanies myoblast fusion (6). In addition, myoferlin can be cleaved by calpain to produce a module consisting of the C2E and C2F domains of myoferlin (40). The C2A domain of myoferlin may bind to phospholipid vesicles, participating in the process of intracellular Ca^2+^ release, as in Ca^2+^-regulated exocytosis (6). Myoferlin had also been shown to interact with VEGFR-2 and to be necessary for correcting VEGF signaling in endothelial cells (41). In addition, myoferlin was found to be involved in gastric cancer resistance to oxaliplatin (42).

Myoferlin has been found to enhance the invasiveness of breast cancer (43), melanoma (44), and pancreatic cancer (45). Myoferlin can mediate the expression of matrix metalloproteinases (MMPs) and epithelial-mesenchymal transition (EMT) (43, 46). Assessment of the function of myoferlin in energy metabolism had shown that myoferlin could modulate the cell metabolism between oxidative phosphorylation and glycolysis, and the conversion of saturated to unsaturated fatty acids in triple-negative breast cancer cells (TNBC) (11). Myoferlin had been found to maintain mitochondrial structure and oxidative phosphorylation in pancreatic cancer and to interact with mitofusin, indicating that myoferlin modulates mitochondrial dynamics, resulting in mitochondrial fusion (14). Prior study had linked mitochondrial fusion/fission to cancer cell metabolism (6). Energy metabolism was crucial for the proliferation and migration of cancer cells (47), with oxidative phosphorylation (OXPHOS) being especially necessary for the migration of cancer cells (45, 48, 49).

ROS were maintained in dynamic balance by reduction-oxidation (redox) reactions in biological system, as well as acting as signaling factor to modulate cellular regulatory pathways (50). Cancer cells were characterized by higher ROS levels than normal cells, with ROS being involved in the activation of proto-oncogenes as well as serving as signaling factors to induce cancer cell growth and metastasis (37). The process of cancer metastasis involved (EMT), which is associated with losses in cell-to-cell adhesion and interactions with the ECM, as well as migration toward and invasion of lymphatic and vascular vessels (51).

ROS had been found to induce Rho family GTPase-dependent cytoskeletal rearrangement, accelerate MMP-dependent ECM protein degradation, and promote hypoxia-inducible factor-dependent angiogenesis, thus participating in the EMT process (37, 52, 53). MYOF had been shown to regulate the expression of MMPs and EMT. Additional studies are needed to determine whether MYOF regulates MMPs by modulating intracellular ROS levels and whether the antioxidant NAC could block the regulatory cascade in gastric cancer cells.

The overexpression of myoferlin in multiple types of cancer (6) suggested that assessment of myoferlin expression may be diagnostic and that this protein could be targeted in cancer treatment. Metabolism is a hallmark of cancer (54), with mitochondria being important in cancer metabolism (55). Because myoferlin has been reported necessary for optimal mitochondrial function, agents targeting myoferlin expression or function may be used in cancer treatment. One possibility is WJ460, a small molecule that directly targets the C2D domain of MYOF (21). WJ460 has been shown reduce breast cancer extravasation into the lung parenchyma *in vivo* (21), and to trigger mitophagy, ROS accumulation and ferroptosis in pancreatic cancer cells (56). Compound 6y, a series of 1,5-diaryl-1,2,4-triazole derivatives, may prevent pancreatic cancer metastasis by targeting MYOF (57) and YQ456, another inhibitor of MYOF, shown a high binding affinity to the C2D domain of MYOF with the significant anti-proliferation and anti-invasion activity in colorectal cancer (13), with the present study showing that NAC could inhibit the MYOF-enhanced migration of cancer cells by reducing intracellular ROS levels. The homeostasis triggered by NAC in MYOF expressing gastric cancer cells suggested tumor-suppressive activity that may be useful clinically in anti-cancer treatment. Additional translational and clinical studies are required to validate this hypothesis.

The present study had several limitations, including its retrospective design. The clinicopathological and prognostic data were from the TCGA-STAD and GEO databases. Patients in these databases received various treatments, including chemotherapy, targeted therapy, and external radiotherapy, differences that may have affected patient OS.

## Conclusions

Disruption of the signaling network between intracellular ROS and myoferlin was associated with reduced migration of gastric cancer cells. NAC targeting ROS modulated by MYOF may be a promising treatment for clinical application.

## Data availability statement

The datasets presented in this study can be found in online repositories. The names of the repository/repositories and accession number(s) can be found in the article/**Supplementary Material**.

## Ethics statement

This study was approved by the Ethics Committee of Tai’an City Central Hospital. All experiments conformed to the ethical principles of the World Medical Association Declaration of Helsinki. Written informed consent was obtained from all participants for their participation in this study.

## Author contributions

Conception and design: XMC and LW. Administrative support: HF and WZL. Provision of study materials: HLS, XY and WZL. Collection and assembly of data: HLS, YYC and QMS. Data analysis and interpretation: HLS and QMS. Manuscript writing: all authors. Final approval of manuscript: all authors.

## Funding

This work was supported by the Shandong Medicine and Health Science Technology Expansion Plan, China [No. 2017WS069] to WZL.

## Conflict of interest

The authors declare that the research was conducted in the absence of any commercial or financial relationships that could be construed as a potential conflict of interest.

## Publisher’s note

All claims expressed in this article are solely those of the authors and do not necessarily represent those of their affiliated organizations, or those of the publisher, the editors and the reviewers. Any product that may be evaluated in this article, or claim that may be made by its manufacturer, is not guaranteed or endorsed by the publisher.

## Supplementary material

Supplementary Figure 1 Effect of *MYOF* overexpression or knockdown on expression of MYOF protein and *MYOF*, *Twist 1*, and *ARPC3* mRNA in gastric cancer cells. **(A)** Western blot assays of MYOF protein levels in HGC27 and SNU1 cells infected with empty vector or vector encoding *MYOF* and in MKN45 cells infected with empty vector or vector encoding sh*MYOF*#1 or sh*MYOF*#2; GAPDH was used as the internal protein loading control. **(B-D)** qRT-PCR assays of the expression of (B) *MYOF*, **(C)** *Twist1* and **(D)** *ARPC3* mRNAs in the cells described in **(A)**. Results are reported as the mean ± SD relative mRNA expression of three independent experiments, using triplicates of each sample in each experiment. *P* values were determined by unpaired two-tailed Student’s *t*-tests or ANOVA. *****P*<0.0001.

Supplementary Figure 2 MYOF promotion of gastric cancer cell motility and intracellular reactive oxygen species (ROS) and motility. **(A, B)** Intracellular ROS levels **(A)** and DCFDA geometric mean fluorescence intensity (MFI) **(B)** of SNU1 gastric cancer cells alone (wild-type) or infected with empty vector or vector encoding *MYOF* treated with vehicle or NAC (5 μM) for 24 h. MFI results are reported as the mean ± SD fold change of three independent experiments compared with SNU1 cells infected with empty vector. **(C, D)** Intracellular ROS levels **(C)** and DCFDA geometric mean fluorescence intensity (MFI) **(D)** of MKN45 cells alone (wild-type) or MKN45 cells alone infected with empty vector or vector encoding sh*MYOF*#1 or sh*MYOF*#2. MFI results are reported as the mean ± SD fold change of three independent experiments compared with MKN45 sh*MYOF*#2 cells. **(E, F)** Intracellular GSH and GSSG levels, expressed as GSH/GSSG ratios **(E),** and intracellular NADPH and NADP+ levels, expressed as NADPH/NADP+ ratios **(F)**, of SNU1 gastric cancer cells alone (wild-type) or infected with empty vector or vector encoding *MYOF* treated with vehicle or NAC (5 μM) for 24 h. Results represent the mean ± SD of three independent experiments. **(G, H)** Intracellular GSH and GSSG levels, expressed as GSH/GSSG ratios **(G),** and intracellular NADPH and NADP+ levels, expressed as NADPH/NADP+ ratios **(H)** of MKN45 cells alone (wild-type) or MKN45 cells alone infected with empty vector or vector encoding sh*MYOF*#1 or sh*MYOF*#2. Results represent the mean ± SD of three independent experiments, with each experiment including triplicate samples. **(I)** Representative images of cell migration assays. Scale bar, 20 μm, 200× magnification. **(J, K)** Statistical analysis of migratory cells. Results are reported as the mean ± SD of three independent experiments, with each experiment including triplicate samples. *P* values were determined using unpaired two-tailed Student’s t-tests or ANOVA. NS, not significant, ****P<0.0001.

## Abbreviations

*MYOF*, myoferlin; GC, gastric cancer; ROS, reactive oxygen species; DMEM, Dulbecco’s modified Eagle’s medium; FBS, fetal bovine serum; cDNA, complementary DNA; qRT-PCR, quantitative real-time PCR; HPA, Human Protein Atlas; DAVID, Database for Annotation, Visualization, and Integrated Discovery; NSE, normalized enrichment score; GSEA, Gene Set Enrichment Analysis; NAC, N-acetyl-L-cysteine; ANOVA, one-way analysis of variance; OS, overall survival; PFS, progression-free survival; PPS, post-progression survival; CCLE, Cancer Cell Line Encyclopedia; TNBC, triple-negative breast cancer cells; EMT, epithelial-mesenchymal transition; ECM, extracellular matrix; TMA, tissue microarray.
