# Supplementary figures and images for: Myoferlin disturbs redox equilibrium to accelerate gastric cancer migration
Source: Front Oncol. 2022 Sep 6;12:905230. doi: 10.3389/fonc.2022.905230 (PMC9486956; doi:10.3389/fonc.2022.905230)

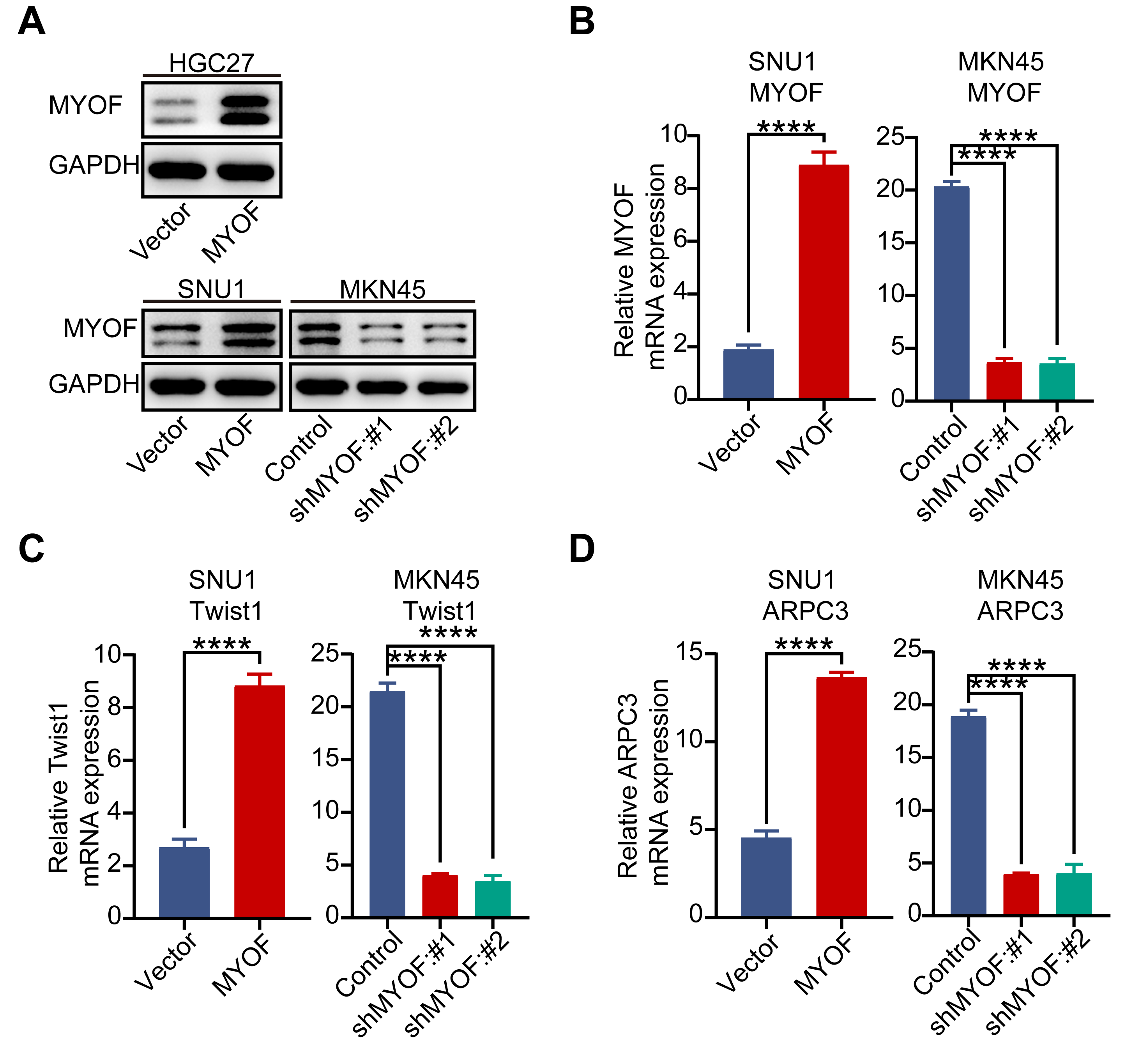

Supplement: Supplementary Figure 1 — Effect of MYOF overexpression or knockdown on expression of MYOF protein and MYOF, Twist 1, and ARPC3 mRNA in gastric cancer cells. (A) Western blot assays of MYOF protein levels in HGC27 and SNU1 cells infected with empty vector or vector encoding MYOF and in MKN45 cells infected with empty vector or vector encoding shMYOF#1 or shMYOF#2; GAPDH was used as the internal protein loading control. (B-D) qRT-PCR assays of the expression of (B) MYOF, (C) Twist1 and (D) ARPC3 mRNAs in the cells described in (A). Results are reported as the mean ± SD relative mRNA expression of three independent experiments, using triplicates of each sample in each experiment. P values were determined by unpaired two-tailed Student’s t-tests or ANOVA. ****P<0.0001. [file Image_1.tif]

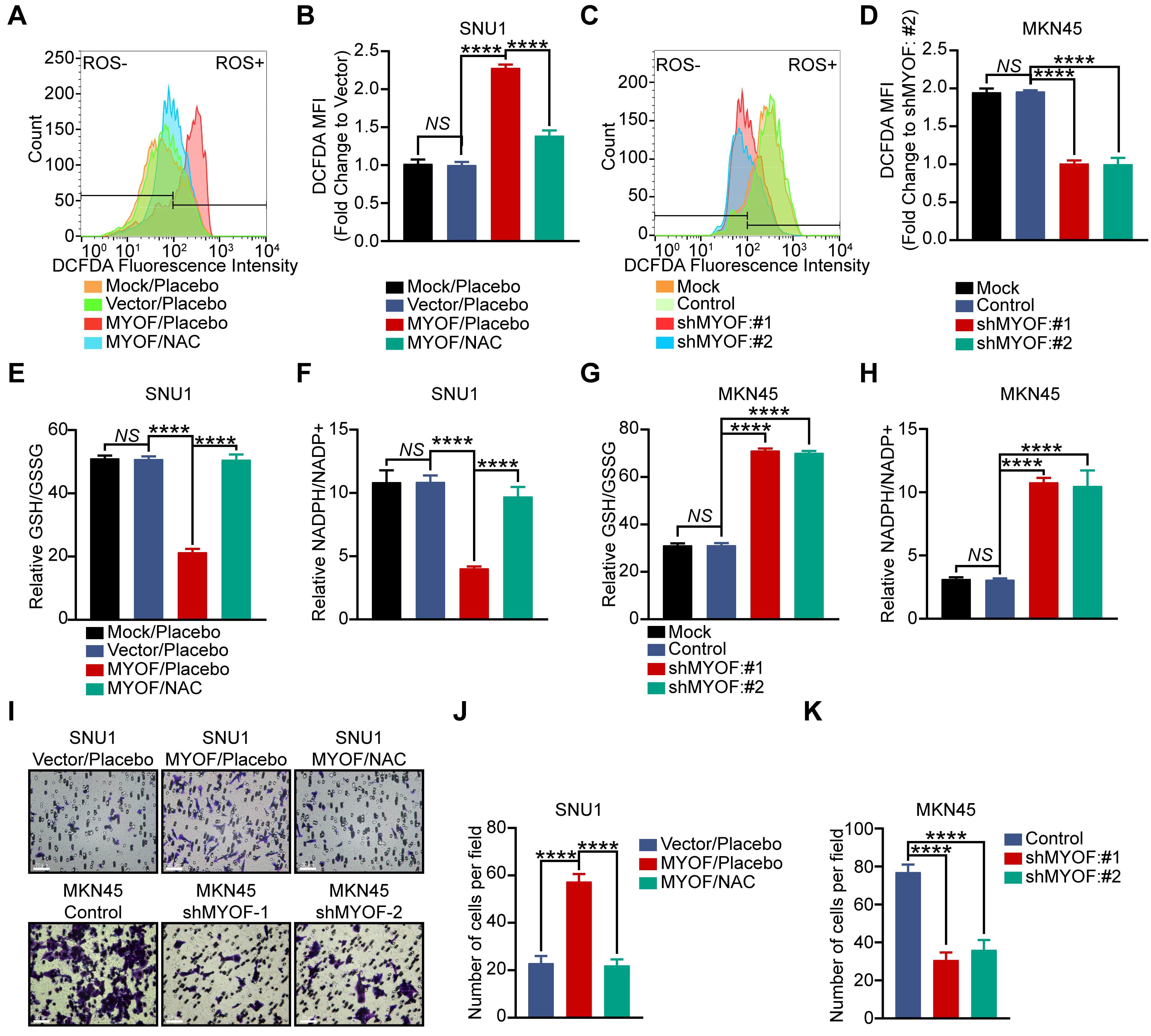

Supplement: Supplementary Figure 2 — MYOF promotion of gastric cancer cell motility and intracellular reactive oxygen species (ROS) and motility. (A, B) Intracellular ROS levels (A) and DCFDA geometric mean fluorescence intensity (MFI) (B) of SNU1 gastric cancer cells alone (wild-type) or infected with empty vector or vector encoding MYOF treated with vehicle or NAC (5 μM) for 24 h. MFI results are reported as the mean ± SD fold change of three independent experiments compared with SNU1 cells infected with empty vector. (C, D) Intracellular ROS levels (C) and DCFDA geometric mean fluorescence intensity (MFI) (D) of MKN45 cells alone (wild-type) or MKN45 cells alone infected with empty vector or vector encoding shMYOF#1 or shMYOF#2. MFI results are reported as the mean ± SD fold change of three independent experiments compared with MKN45 shMYOF#2 cells. (E, F) Intracellular GSH and GSSG levels, expressed as GSH/GSSG ratios (E), and intracellular NADPH and NADP+ levels, expressed as NADPH/NADP+ ratios (F), of SNU1 gastric cancer cells alone (wild-type) or infected with empty vector or vector encoding MYOF treated with vehicle or NAC (5 μM) for 24 h. Results represent the mean ± SD of three independent experiments. (G, H) Intracellular GSH and GSSG levels, expressed as GSH/GSSG ratios (G), and intracellular NADPH and NADP+ levels, expressed as NADPH/NADP+ ratios (H) of MKN45 cells alone (wild-type) or MKN45 cells alone infected with empty vector or vector encoding shMYOF#1 or shMYOF#2. Results represent the mean ± SD of three independent experiments, with each experiment including triplicate samples. (I) Representative images of cell migration assays. Scale bar, 20 μm, 200× magnification. (J, K) Statistical analysis of migratory cells. Results are reported as the mean ± SD of three independent experiments, with each experiment including triplicate samples. P values were determined using unpaired two-tailed Student’s t-tests or ANOVA. NS, not significant, ****P<0.0001. [file Image_2.tif]
